# Is It Personal? The Effect of Personal vs. Occupational Trauma on PTSD Symptom Severity in Emergency Responders

**DOI:** 10.3389/fpsyt.2022.856895

**Published:** 2022-06-17

**Authors:** Jennifer Wild, Tingyee E. Chang

**Affiliations:** ^1^Department of Experimental Psychology, University of Oxford, Oxford, United Kingdom; ^2^Department of Emergency Medicine, New York University (NYU) School of Medicine, New York, NY, United States

**Keywords:** PTSD, trauma, first responders, emergency responders, criterion A, police, paramedics, firefighters

## Abstract

Emergency responders are exposed to potentially traumatic events in their line of work and as such, are at increased risk of developing post-traumatic stress disorder (PTSD). Little is known about the characteristics of trauma associated with PTSD symptoms in this population. This study analyzed the self-reported worst traumatic event on the PTSD checklist for DSM-5 for a sample of *N* = 610 emergency responders, working as police officers, paramedics, firefighters or search and rescue personnel. Sufficient information was available to code 98% (*N* = 603) participants' trauma; 84% (*N* = 509) met DSM-V criterion A trauma. Of the participants reporting criterion A trauma, 56.9% (*N* = 290) participants reported being most affected by a traumatic event that occurred in their personal lives, 41.5% (*N* = 211) participants reported being most affected by a work-related traumatic event and 1.6% (*N* = 8) reported criterion A events that were work-related and had occurred prior to their role as an emergency responder (e.g., combat). Paramedics were significantly more likely to report occupational trauma as their worst event whereas police officers, firefighters, and search and rescue workers reported personal trauma as their worst event. Personal trauma was associated with significantly greater PTSD symptom severity than occupational trauma. Emergency responders identifying as women were significantly more likely to report personal than work-related trauma as their index event and men were more likely to report work-related than personal trauma as being linked to their PTSD symptoms. The results underscore the need to consider the broader context of trauma in the emergence of PTSD symptoms in emergency workers.

## Introduction

Whilst numerous studies document high rates of post-traumatic stress disorder (PTSD) in emergency responders (1, 2), little is known about the traumatic events associated with symptoms. Trauma exposure leading to PTSD in this population is often thought to have been experienced at work. However, new research suggests that for frontline emergency workers, non-occupational trauma, such as interpersonal violence or traumatic bereavement, occurring in their personal lives, may be more significant than occupational trauma, such as intervening in a road traffic accident or cardiac arrest. Among frontline healthcare staff working during the peak of the COVID-19 pandemic, for example, rates of PTSD were found to be high yet contrary to hypothesis, index events associated with the diagnosis were reported to have occurred in staff's personal lives as often as they were reported to have occurred at work (3). Similarly, in military samples, non-occupational trauma is reported to be highly prevalent and to increase risk for PTSD (4). For first responders, it is critical to disentangle the nature of trauma associated with PTSD for service provision and treatment planning, and to better understand symptom development and persistence. Among emergency workers, it is unclear to what extent trauma associated with PTSD symptom severity relates to exposure at work or in their personal lives, whether the experience of trauma encountered outside of work may be more problematic than occupational trauma exposure, and whether there are any differences in trauma exposure between service groups or gender.

Police are reported to rate as highly stressful a wide range of potentially traumatic occupational events, such as intentional injury of another person or witnessing the death of a colleague (5), both of which are less commonly experienced by paramedics and firefighters. Research focused on firefighters reports distressing personal experiences to be robust predictors of PTSD symptoms (6). Other studies have found paramedics to experience high rates of exposure to occupational trauma associated with longer contact with injured people and medical interventions with life and death consequences than police and firefighters (7). Paramedics also report high rates of childhood trauma (8), which relate to early adverse events in their personal lives. Firefighters and paramedics identifying as women are reported to be more likely to develop PTSD than men (2, 9), which is consistent with epidemiological research of PTSD (10).

Uptake of occupational support services amongst first responders is typically poor (11) and this is despite high rates of PTSD (1) as well as, amongst paramedics, high rates of suicide (12). Research has identified that one of the core contributors to the poor uptake of support services is stigma and fear of embarrassment (13–15). Knowing that trauma occurring in first responders' personal lives may be prevalent and for many, may be equally or even more problematic than occupational trauma in terms of PTSD symptomatology, is significant for two reasons. First, such a finding would have implications for improving uptake of support services. The occurrence of personal trauma and associated difficulties could be normalized in efforts to reduce stigma. Awareness campaigns could focus messages on normalizing the range of trauma experienced, the importance of recognizing symptoms and that such experiences are common both at and outside of work. Research suggests that the normalization of symptoms predicts good outcome (16). Thus, raising awareness that first responders may experience exposure to potentially traumatic events at work or outside of work and may develop symptoms in response to either normalizes these experiences, may proffer some symptom relief and importantly, encourage help-seeking. Second, such knowledge underscores the importance of investigating the interplay between trauma exposure and PTSD in this group. It is unclear whether repeated exposure to occupational trauma makes it more difficult to deal with trauma in one's personal life or vice versa in this population. Research suggests that there may be a tipping point at which first responders experience greater risk for PTSD with each additional trauma exposure (16).

It is clear that trauma occurring in one's personal life is likely to influence this tipping point. In this study, we analyze the Post-Traumatic Stress Disorder Checklist (PCL-5) (17) and linked index event of a large sample of emergency responders working as police officers, paramedics, firefighters or search and rescue personnel. Given the literature attributing high rates of PTSD among emergency services to occupational trauma exposure, we hypothesized that emergency responders would be more likely to identify as their worst event an occupational rather than personal trauma, that the different emergency services (i.e., police, paramedic, firefighter and search and rescue) would report similar rates of personal and work-related traumatic events and that there would be no difference in PTSD symptom severity between occupational and personal trauma. Given that women are at greater risk of lifetime sexual assault trauma than men, we hypothesized that women would be more likely to identify trauma in their personal lives as their worst event.

## Methods

### Participants

Police officers, firefighters, paramedics and search and rescue personnel were invited to complete the PCL-5, which first included questions on trauma exposure and an instruction to complete the questionnaire in relation to the worst event the participant had identified. Participants completed the PCL-5 prior to random allocation in one of two randomized controlled trials evaluating a free resilience intervention (18, 19). The first trial recruited first responders across 9 sites in England; the resilience course was delivered over 6 weeks for 2 h a week. The second trial recruited at 5 sites across England; the course was delivered over 4 weeks, also 2 h per week. For both studies, recruitment methods involved giving talks at emergency service sites, circulating emails, posters, and leaflets, and using social media. In the first study, *N* = 151 did not reply after signing up, leaving a total sample size of *N* = 430. In the second study, *N* = 63 did not reply after signing up, leaving a total sample size of *N* = 180. The combined sample consisted of *N* = 610 participants. Informed consent was obtained electronically in accordance with ethical approval given by the University of Oxford's Medical Sciences Interdivisional Research Ethics Committee (MS-IDREC-C1-2015-059; MS-IDREC- R47862/RE002). Here we report on the index events associated with the PCL-5. These data have not previously been reported.

### Measures

Participants completed a number of measures through a secure digital platform. Questionnaires were completed at baseline, post-intervention and at follow-up to the resilience intervention, standard practice or placebo control. For the purposes of this study, we report on the PCL-5 completed at baseline prior to the intervention.

#### Demographic Questionnaire (Unpublished)

This questionnaire records information on age, gender, ethnicity, and relationship status. Questions regarding gender asked whether the first responder identified as male, female, other or prefer not to say.

#### Post-traumatic Stress Disorder Checklist (PCL-5)

The PCL-5 (17) is a 20-item questionnaire that parallels the diagnostic criteria for PTSD established in the fifth edition of the Diagnostic and Statistical Manual for Mental Disorders (DSM-5) (20). Items are rated on a scale of “0 = not at all” to “4 = extremely”. The questionnaire first defines a very stressful experience, for example an experience involving actual or threatened death, serious injury or sexual violence that could be experienced directly or witnessed or learned about happening to a close friend or family member. The questionnaire then asks the participant to identify their worst event and to answer subsequent questions in relation to their identified event. Total scores on the PCL-5 range from 0 to 84. A score of 31 or above suggests probable PTSD (17). Internal reliability of the scale in our sample was excellent, Cronbach's alpha = 0.95.

### Analyses

The text descriptions of the PCL-5 were coded to determine whether the reported index event met criterion A. Then the events were categorized according to whether or not they occurred at work or in one's personal life. Thirty percent of coded events were re-rated by an independent assessor and kappa reliability was calculated.

Trauma was determined to meet criterion A if the question relating to threat on the PCL-5 was endorsed. The question asked if the event involved actual or threatened death, serious injury or sexual violence.

Trauma was rated as being occupational if it clearly occurred at work. Indications of work-related trauma included references to treating patients or trauma involving colleagues. Coders were asked to rate trauma that occurred outside of work as personal trauma. Indications of personal-related trauma included references to family, to friends or details surrounding trauma that one had encountered personally, such as life-threatening illness or childhood sexual or physical abuse.

Occupational examples include: witnessing a suicide in which a patient jumped from a balcony, being first on scene to a road traffic collision, which resulted in the death of a teenager, attending a suicide in which a patient had shot himself, being the first person on scene to a cardiac arrest of a baby, being stabbed at work on a call-out by a dirty needle in which the offender claimed to be HIV positive.

Examples of personal trauma include: death of a close friend, being threatened by ex-partner in public with a knife, being diagnosed with a rare illness, sudden death of spouse, accident leading to participant's child having a serious head injury, victim of physical violence growing up.

When responses were coded as “uncertain”, the two coders met and consulted the question on the PCL-5 which refers to how the trauma was experienced. If the trauma was experienced through repeated exposure at work, then the index event was coded as occupational. There were few (*N* = 14) instances where on initial coding, the index trauma was rated as uncertain. All of these were reported as having been experienced through repeated exposure as part of the job and as such, were rated as being occupational.

Chi square analysis was conducted to evaluate the first hypothesis that emergency workers would be more likely to report an occupational rather than a personal trauma as their worst event with no differences among the different services (i.e., police, paramedic, fire and search, and rescue services) and the second hypothesis that there would be a relationship between trauma type (occupational vs. personal) and gender (male vs. female). To analyze PTSD symptom severity by trauma type and service, independent samples *t-*tests were conducted.

## Results

### Participants

The total sample consisted of 610 emergency service workers (57% female, 90.5% White British, 7.4% Black Asian Minority Ethnic, mean_age_ = 41.74, *SD*_age_ = 9.48) who worked in roles as police officers (*N* = 368; 60.3%), paramedics (*N* = 135; 22.1%), firefighters (*N* = 90, 14.8%) and search and rescue personnel (*N* = 17; 2.8%). Table 1 shows the demographic characteristics of the sample for each of the four emergency service roles.

**Table 1:** Participant demographic characteristics.

	**Police** **(*n* = 368)**	**Paramedics** **(*n* = 135)**	**Firefighters** **(*n* = 90)**	**Search and rescue** **(*n* = 17)**	**Total** **(*n* = 610)**
% Female	62.5	50.4	51.1	52.9	57.8
Mean age (SD)	41.83 (9.1)	39.51 (9.86)	44.70 (8.71)	41.82 (13.70)	41.74 (9.48)
**Marital status** ***n*** **(%)**					
Single/living alone	69 (18.6)	29 (21.5)	12 (13.3)	3 (17.6)	113 (18.5)
Married/living together	258 (70.1)	89 (65.9)	65 (72.2)	14 (82.4)	426 (69.8)
Divorced/widowed/separated	39 (10.6)	17 (12.6)	13 (14.4)	0	69 (11.3)
Other	2 (0.54)	0	0	0	2 (0.33)
**Ethnicity** ***n*** **(%)**					
White British/European	330 (89.7)	131 (97.0)	87 (96.7)	17 (100)	565 (92.6)
Black/Indian/Asian/Arab/Other	38 (10.3)	4 (3.0)	3 (3.3)	0	45 (7.4)

### Criterion A and Occupational vs. Personal Trauma

Of the 610 participants, sufficient information was available to code *N* = 603 participants' (98%) trauma. Of these trauma, 509 (84%) met criterion A of the DSM-V PTSD diagnosis. Of the participants reporting criterion A trauma, *N* = 290 (56.9%) participants reported being most affected by a criterion A event (e.g., interpersonal violence and death of a loved one) that occurred in their personal lives, *N* = 211 (41.5%) participants reported being most affected by a work-related traumatic event (e.g., death and transport accident) and *N* = 8 (1.6%) reported criterion A events that were work-related and had occurred prior to their role as an emergency responder (e.g., combat). A random sample of 30% of index events were rated by an independent assessor as to whether they were occupational or personal in nature, demonstrating excellent agreement (Cohen's κ = 0.989). Chi square analysis indicated a significant association between trauma type and service with police officers, firefighters, and search and rescue workers being significantly more likely to report personal trauma, while paramedics were more likely to report occupational trauma as their worst event, *X*^2^ (3, *N* =501) = 10.78, *p* = 0.01, Cramer's *V* = 0.15. Table 2 reports how the trauma had been experienced, such as directly, by witnessing it or through repeated exposure at work. Table 3 details the frequency of occupational vs. personal trauma by service and Table 4 illustrates the type of trauma (e.g., interpersonal violence, traumatic childbirth, and death trauma) experienced by participants and endorsed as their worst event.

**Table 2:** How the criterion A index trauma was experienced.

	**Criterion A trauma** ***n*** **(%)**			
	**Occupational** **(*n* = 211)**	**Personal** **(*n* = 290)**	**Other (i.e., combat)** **(*n* = 8)**	**Total**
Experienced directly	44 (20.9)	139 (47.9)	4 (50)	187 (36.7)
Witnessed	69 (32.7)	76 (26.2)	1 (12.5)	146 (28.7)
Learning about the event happening to a close family member or friend	2 (0.9)	54 (18.6)	0 (0)	56 (11.0)
Repeated exposure at work	94 (44.5)	0 (0)	3 (37.5)	97 (19.1)
Other	1 (0.5)	21 (7.2)	0 (0)	22 (4.3)
Missing	1 (0.5)	0 (0)	0 (0)	1 (0.2)
Total	211	290	8	509

**Table 3:** Criterion A index trauma by service.

	**Police** **(*n* = 310)**	**Paramedics** **(*n* = 120)**	**Firefighters** **(*n* = 66)**	**Search and rescue** **(*n* = 13)**	**Total** **(*n* = 509)**
**Criterion A index trauma** ***n*** **(%)**					
Occupational	121 (39.0)	64 (53.3)	22 (33.33)	4 (30.8)	211 (41.5)
Personal event	186 (60.0)	53 (44.17)	42 (63.64)	9 (69.2)	286 (56.19)
Other occupational (i.e., combat)	3 (0.97)	3 (2.5)	2 (3.03)	0	8 (1.6)

**Table 4:** Criterion A index trauma type.

	**Occupational** **(*n* = 211)**	**Personal** **(*n* = 290)**	**Prior occupational** **(e.g., combat) (*n* = 8)**	**Total** **(*n* = 509)**
**Criterion A trauma type** ***n*** **(%)**				
Death trauma	90 (42.7)	143 (49.3)	1 (12.5)	243 (47.7)
Road traffic accident	48 (22.7)	26 (9.0)	0	74 (14.5)
Severe physical illness	23 (10.9)	49 (16.9)	0	72 (14.1)
Interpersonal violence	20 (9.5)	29 (10.0)	0	49 (9.6)
Childhood abuse	7 (3.3)	14 (4.8)	1 (12.5)	22 (4.3)
Sexual assault	3 (1.4)	16 (5.5)	2 (25.0)	21 (4.1)
Fire	13 (6.2)	3 (1.0)	0	16 (3.1)
Traumatic childbirth	1 (0.5)	8 (2.8)	0	9 (1.8)
Combat	0	1 (0.3)	4 (50.0)	5 (1.0)
Natural disaster	4 (1.9)	0	0	4 (0.8)
Terrorist attack	2 (0.9)	1 (0.3)	0	3 (0.6)

Chi square analysis indicated a significant association between trauma type and gender with women being more likely to report criterion A trauma experienced in their lives outside of work whilst men were more likely to report occupational trauma as their worst event, *X*^2^ (1, *N* = 501) = 53.82, *p* = 0.001, Cramer's *V* = 0.33. Of the *N* = 292 women reporting criterion A trauma, *N* = 209 (71.5%) reported personal trauma and *N* = 83 (28.4%) reported occupational trauma whilst of the *N* = 211 men reporting criterion A trauma, *N* = 128 (60.7%) reported occupational trauma and *N* = 81 (38.4%) reported personal trauma as their worst event.

### PTSD Symptom Severity

Criterion A trauma that emergency services personnel experienced in their personal lives was associated with greater PTSD symptom severity (mean PCL-5 score = 10.77, SD = 13.14) compared to trauma experienced at work (mean PCL-5 score = 5.51, SD = 8.85), [*t*_(492.47)_ = 5.03, *p* < 0.001]. Of participants endorsing criterion A trauma, *N* = 37 (7.3%) scored above clinical cut-off on the PCL-5, suggesting probable PTSD. Of these participants, *N* = 32 (86.5%) endorsed personal trauma and *N* = 5 (13.5%) endorsed occupational trauma.

For occupational trauma, there were no differences in PTSD symptom severity between subtypes of trauma. For trauma that occurred in one's personal life, there were, however, significant differences in PTSD symptom severity between trauma subtypes [*F*_(7, 284)_ = 2.27, *p* = 0.029] with interpersonal violence, sexual assault, and childhood abuse accounting for significantly greater PTSD symptom severity than other subtypes.

## Discussion

This study analyzed the index events associated with PTSD symptom severity for a large sample of emergency workers. Contrary to prediction, more than half of the sample identified criterion A trauma experienced in their personal lives as their worst event and this was most likely to be true for police officers, firefighters and search and rescue personnel whereas paramedics were significantly more likely to endorse occupational trauma as their worst event. This is perhaps unsurprising given that the nature and frequency of exposure to death and physical injury is higher for paramedics than other emergency occupations (7). Individuals endorsing personal trauma as their index event experienced greater PTSD symptom severity than individuals who identified occupational trauma as their worst event with scores falling in the mild range for the former and minimal range for the latter. Seven percent of the sample met criteria for probable PTSD and their index trauma was significantly more likely to have occurred in their personal lives than at work. Consistent with Olff et al. (10), women were more likely to report a criterion A index event in their personal lives than men who typically endorsed an occupational trauma as their worst event. These results are in line with research which identifies trauma experienced outside of work as being commonly associated with PTSD in at risk occupations and underscores the need to assess index events when investigating PTSD symptomatology (3).

This study identified that a large proportion of personal trauma was experienced directly and a similarly large proportion of occupational trauma was experienced through repeated exposure as part of the job. It should be noted that there may be differences in how participants interpreted how they experienced their trauma, particularly for occupational events. For example, participants often coded “being the first officer on the scene of an RTA” as “It happened directly to me” whereas other participants reported “first on scene to an RTA job” as “I witnessed it”. First responders were likely to have experienced or witnessed the aftermath of RTAs rather than experiencing or witnessing the RTAs themselves. Thus, either category could capture the first responder's experience and will be influenced by how they interpret this question on the PCL-5.

That personal trauma is prevalent and associated with greater symptomatology is perhaps unsurprising given the role negative appraisals of trauma and sequelae bear in the onset and persistence of PTSD symptoms (21, 22). Negative appraisals associated with trauma may exact a more enduring nature when the event takes place in one's personal life where there may be less support in place or limited access to others who have been through similar trauma and whose experiences could help to normalize symptoms and thoughts. The association between negative appraisals and symptoms in this sample is unclear, however, since appraisals were not measured. Of note, interpersonal violence was one of the more commonly experienced personal trauma in this sample and consistent with the literature was associated with greater PTSD symptom severity (23). Childhood abuse and sexual assault were also associated with greater PTSD symptom severity consistent with published studies (23, 24). These trauma may be relevant to Complex PTSD, which can develop in response to childhood trauma as well as repeated exposure to trauma, and is increasingly being diagnosed among police officers (24) and firefighters (25) although it should be noted that we did not measure complex PTSD in this sample.

Our results suggest that exposure to criterion A trauma is high with 84% of the sample reporting a worst event which met this criterion, which is greater than the general population rate of 70% reported in epidemiological surveys (26). Whilst emergency responders appear to be at risk of trauma exposure encountered on-the-job and outside of work, probable PTSD was more likely to be linked to trauma experienced outside of work. This finding is worth bearing in mind when planning and supporting access to services, particularly in-house services for this population. Stigma is often reported as a barrier to help-seeking in high risk occupations (13–15). Normalizing the full range of trauma that first responder occupations are likely to face may help to reduce stigma and improve uptake of services. Future research could investigate the efficacy of organizational messaging to reduce stigma and the potential relationship to service uptake. Importantly, the interplay between trauma exposure and PTSD in this population requires further research. There appears to be a tipping point related to trauma exposure and the onset of PTSD (27). It is likely that exposure to personal trauma influences this tipping point. With reference to occupational trauma, it should be noted that whilst emergency workers do indeed cite such trauma as being problematic for their mental health, they also weight organizational pressures almost equally in terms of negative mental health consequences (28). Although research suggests a relationship between length of service and PTSD symptoms (24, 27) not all studies support this finding (29). It is conceivable that greater years of service exposes emergency responders to a greater number of trauma, both at and outside of work, and may contribute to PTSD onset.

Whilst our results help to elucidate the nature of trauma perceived to be worst events for emergency workers, there are several limitations worth considering. One limitation was that few search and rescue personnel participated in the research and as such, our results may not generalize to the broader profession. Our sample was self-selected. As such rates of trauma exposure and PTSD symptoms may not reflect the wider community of emergency responders. However, our sample was large and the overall rate of probable PTSD is consistent with published studies in these populations (1, 30). Probable PTSD was ascertained by referring to recommended clinical cut-offs for the PCL-5. This method may under-estimate the true rate of PTSD since studies administering both the PCL-5 and structured clinical interviews have found higher rates through interview than *via* self-report questionnaire (3). Another limitation is that index events were not assessed by interview. There may be discrepancies between self-report and clinician assessment of index traumatic events. To reduce over-estimating the frequency of criterion A trauma, only trauma that the participant had endorsed on the PCL-5 as involving actual or threatened death, serious injury or sexual violence was considered to meet criterion A. To ascertain the reliability of the classification of occupational vs. personal trauma, inter-rater reliability was calculated and was excellent. Additionally, none of the trauma that was coded as having taken place in one's personal life was rated on the PCL-5 as having been experienced through repeated exposure at work lending support to the reliability of the classification of participants' trauma. Finally, we did not measure cumulative exposure to trauma. We are unable to draw conclusions on possible relationships between cumulative occupational or personal trauma exposure. That is, cumulative trauma exposure at work may make it more difficult to cope with trauma in one's personal life or vice versa. Nonetheless, our research underscores the importance of considering the broad range of trauma first responders face in their lives in order to better understand the mental health needs of this population.

## Data Availability Statement

The raw data supporting the conclusions of this article will be made available by the authors, without undue reservation.

## Ethics Statement

The studies involving human participants were reviewed and approved by University of Oxford's Medical Sciences Interdivisional Research Ethics Committee (MS-IDREC-C1-2015-059 and MS-IDREC-R47862/RE002). The patients/participants provided their informed consent to participate in this study electronically.

## Author Contributions

JW conceptualized and designed the study and wrote the first draft of the manuscript. TC organized the database. JW and TC conducted the data analyses. All authors contributed to the article and approved the submitted version.

## Funding

This research was supported by Mind CQR01090 awarded to JW. JW's research is supported by MQ (CQRO1260), the Wellcome Trust (00070), and the Oxford Health NIHR Biomedical Research Center.

## Conflict of Interest

The authors declare that the research was conducted in the absence of any commercial or financial relationships that could be construed as a potential conflict of interest.

## Publisher's Note

All claims expressed in this article are solely those of the authors and do not necessarily represent those of their affiliated organizations, or those of the publisher, the editors and the reviewers. Any product that may be evaluated in this article, or claim that may be made by its manufacturer, is not guaranteed or endorsed by the publisher.

## References

[1] Berger W Countinho ESF Figueira I Marques-Portella C Luz MP Neylan TC. Rescuers at risk: a systematic review and meta-regression analysis of the worldwide current prevalence and correlates of PTSD in rescue workers. Soc Psychiatry Psychiatr Epidemiol. (2012) 47.6:1001–11. 10.1007/s00127-011-0408-2 21681455 PMC3974968

[2] Soravia LM Schwab S Walther S Muller. Rescuers at risk: posttraumatic stress symptoms among police officers, fire fighters, ambulance personnel, and emergency and psychiatric nurses. Front Psychiatry. (2021) 11:602064. 10.3389/fpsyt.2020.602064 33542696 PMC7851799

[3] Wild J McKinnon A Wilkins A Browne H. Posttraumatic stress disorder and major depression among frontline healthcare staff working during the COVID-19 pandemic. Br J Clin Psychol. (2021). 10.1111/bjc.12340. [Epub ahead of print]. 34713436 PMC8646304

[4] Clancy CP Graybeal A Tompson WP Badgett KS Feldman ME Calhoun PS. Lifetime trauma exposure in veterans with military-related posttraumatic stress disorder: association with current symptomatology. J Clin Psychiatry. (2006) 67:1346–53. 10.4088/JCP.v67n0904 17017820

[5] Violanti JM Aron F. Ranking police stressors. Psychol Rep. (1994) 75:824–6. 10.2466/pr0.1994.75.2.824 7862790

[6] Morren M Yzermans CJ van Nispen RM Wevers SJ. The health of volunteer firefighters three years after a technological disaster. J Occup Health. (2005) 47:523–32. 10.1539/joh.47.523 16369116

[7] Marmar CR Weiss DS Metzler TJ Ronfeldt HM Foreman C. Stress responses of emergency services personnel to the Loma Prieta earthquake Interstate 880 freeway collapse and control traumatic incidents. J Trauma Stress. (1996) 9:63–85. 10.1002/jts.2490090107 8750452

[8] Maunder RG Halpern J Schwartz B Gurevich M. Symptoms and responses to critical incidents in paramedics who have experienced childhood abuse and neglect. Emerg Med J. (2012) 29:222–7. 10.1136/emj.2010.099838 21422029

[9] Noor N Pao C Dragomir-Davis M Tran J Arbona C. PTSD symptoms and suicidal ideation in US female firefighters. Occup Med (Lond). (2019) 69:577–85. 10.1093/occmed/kqz057 31064010

[10] Olff M Langeland W Draijer N Gersons BPR. Gender differences in posttraumatic stress disorder. Psychol Bull. (2007) 133.2:183–204. 10.1037/0033-2909.133.2.183 17338596

[11] Wild J Greenberg N Moulds ML Sharp ML Fear N Harvey S. Pre-incident training to build resilience in first responders: recommendations on what to and what not to do. Psychiatry. (2020) 83:128–42. 10.1080/00332747.2020.1750215 32338579

[12] Vigil NH Grant AR Perez O Blust RN Chikani V Vadeboncoeur TF. Death by suicide-the EMS profession compared to the general public. Prehosp Emerg Care. (2019) 23:340–5. 10.1080/10903127.2018.1514090 30136908

[13] Johnston S Wild J Sanderson K Kent B. Perceptions and experiences of employee mental health support: a cross-sectional survey of a large ambulance Trust in the UK. J Paramed Pract. (2022) (In press).

[14] Haugen PT McCrillis AM Smid GE Nijdam MJ. Mental health stigma and barriers to mental health care for first responders: a systematic review and meta-analysis. J Psychiatr Res. (2017) 94:218–29. 10.1016/j.jpsychires.2017.08.001 28800529

[15] Auth NM Booker MJ Wild J Riley R. Mental health and help seeking among trauma-exposed emergency service staff: a qualitative evidence synthesis. BMJ Open. (2022) 12:e047814. 10.1136/bmjopen-2020-047814 35110304 PMC8811562

[16] Dudley R Bryant C Hammond K Siddle R Kingdon D Turkington D. Techniques in cognitive behavioural therapy: using normalising in schizophrenia. Psykologi. (2007). Available online at: https://psykologtidsskriftet.no/2007/05/techniques-cognitivebehavioural-therapy-using-normalising-schizophrenia (accessed May 4, 2022).

[17] Weathers FW Litz BT Keane TM Palmieri PA Marx BP Schnurr PP. The PTSD Checklist for DSM-5 (PCL-5). Scale available from the National Center for PTSD. (2013). Available online at: www.ptsd.va.gov (accessed January 17, 2022).

[18] Wild J El-Salahi S Degli Esposti M Thew GR. Evaluating the effectiveness of a group-based resilience intervention versus psychoeducation for emergency responders in England: a randomised controlled trial. PLoS ONE. (2020) 15:e0241704. 10.1371/journal.pone.0241704 33180798 PMC7660584

[19] Wild J Tyson G Beier E Degli Esposti M. Cognitive Training in Resilience for Emergency Responders Versus Psychoeducation and Standard Practice: A Randomized Controlled Trial. (under review).

[20] American Psychiatric Association. Diagnostic and Statistical Manual of Mental Disorders. 5th ed. Washington, DC: American Psychiatric Association (2013).

[21] Dunmore E Clark DM Ehlers A. A prospective investigation of the role of cognitive factors in persistent posttraumatic stress disorder (PTSD) after physical or sexual assault. Behav Res Ther. (2001) 39:1063–84. 10.1016/S0005-7967(00)00088-7 11520012

[22] Halligan SL Michael T Clark DM Ehlers A. Posttraumatic stress disorder following assault: the role of cognitive processing, trauma memory, and appraisals. J Consult Clin Psychol. (2003) 71:419–31. 10.1037/0022-006X.71.3.419 12795567

[23] Kessler RC Aguilar-Gaxiola S Alonso J Benjet C Bromet EJ. Trauma and PTSD in the WHO World Mental Health Surveys. Eur J Psychotraumatol. (2017) 8.5:1353383. 10.1080/20008198.2017.1353383 29075426 PMC5632781

[24] Brewin CR Miller JK Soffia M Peart A Burchell B. Posttraumatic stress disorder and complex posttraumatic stress disorder in UK police officers. Psychol Med. (2022) 52:1287–95. 10.1017/S0033291720003025 32892759

[25] Langtry J Owczarek M McAteer D Taggart L Gleeson C Walshe C. Predictors of PTSD and CPTSD in UK firefighters. Eur J Psychotraumatol. (2021) 12:1849524. 10.1080/20008198.2020.1849524 33680343 PMC7874934

[26] Benjet C Bromet E Karam EG Kessler RC McLaughlin KA Ruscio AM. The epidemiology of traumatic event exposure worldwide: results from the World Mental Health Survey Consortium. Psychol Med. (2016) 46:327–43. 10.1017/S0033291715001981 26511595 PMC4869975

[27] Harvey SB Milligan-Saville JS Paterson HM Harkness EL Marsh AM Dobson M. The mental health of fire-fighters: an examination of the impact of repeated trauma exposure. Austr N Zeal J Psychiatry. (2016) 50:649–58. 10.1177/0004867415615217 26607303

[28] Mind Blue Light Programme. Blue Light Scoping Survey. Mind (2015). Available online at: https://www.mind.org.uk/media-a/4586/blue-light-scoping-survey-sar-final.pdf (accessed January 17, 2022).

[29] Streb M Haller P Michael T. PTSD in paramedics: resilience and sense of coherence. Behav Cogn Psychother. (2014) 42:452–63. 10.1017/S1352465813000337 23714206

[30] Klimley KE Van Hasselt VB Stripling AM. Posttraumatic stress disorder in police, firefighters, and emergency dispatchers. Aggress Violent Behav. (2018) 43:33–44. 10.1016/j.avb.2018.08.005

